# Repeated implant analog reuse effect on dimensional accuracy and marginal gap with abutments - An *in vitro* study

**DOI:** 10.6026/973206300212050

**Published:** 2025-07-31

**Authors:** Nilanjana Majumdar, Poonam Agrawal, Rahulkumar R. Patel, Siraj Uddin Khan, Subasish Behera, Soumyaranjan Nanda, Miral Mehta

**Affiliations:** 1 Department of Periodontology & Oral Implantology, Kalinga Institute of Dental Sciences, KIIT deemed to be University, Bhubaneswar, Odisha, India; 2 Deparment of Periodontology, Dental Surgeon, Government of Odisha, CHC- Ranpur, Nayagarh, Odisha, India; 3 Department of Prosthodontics, Crown and Bridge, Vaidik Dental College and Research Centre, Daman; 4 Department of Prosthodontics and Crown and Bridge, Maitri College of Dentistry and Research Center, Durg, Chhattisgarh, India; 5 Department of Conservative Dentistry and Endodontics, Government Dental College and Hospital (SCB), Manglabag, Cuttack, Odisha, India; 6 Private Practitioner, Cuttack, Odisha, India; 7 Department of Pediatric and Preventive Dentistry, Karnavati School of Dentistry, Karnavati University, Gandhinagar, Gujarat, India

**Keywords:** Implant analog, abutment fit, marginal gap, dimensional accuracy, reuse cycles, prosthodontics, *in vitro* study

## Abstract

The use of same implant analog multiple times affects their accuracy and the distance between the implant and abutment. Hence, 30 titanium analogs were grouped according to how many patients had worn them before (0, 5 and 10). Stereomicroscopy and digital calipers were used to measure both the marginal gap and dimensional changes. The evidence points to clearly increased differences and dimensional losses as the parts were used and reused (p < 0.001).

## Background:

Prosthodontists use implant analogs to take on the role of dental implants, especially when creating prosthetic restorations. Because these components are made to match the dental implant in size, shape and interface, the intraoral implant location can be accurately transferred to the working cast [1]. Simulating the implant-abutment fit on the working model will help guarantee a passive fit, proper function and long-term success for the prosthesis. This is most crucial when there are several implants or prostheses being used in one treatment [2]. It is common in clinical and laboratory settings to reuse implant analogs for many prosthetic fabrications when resources are limited. Using the same components can reduce costs and prevent waste, yet there is a risk that while tightening, removing, or cleaning them, they may be damaged and worn out [3, 4]. With time, mechanical loads such as biting, shaping and handling can make the analog shift in size, most noticeably at the connection where it joins the abutment [5]. Although these changes are not easily spotted, they can have a major impact on the accuracy of prosthesis production and the success of its treatment. To be successful for a long time, the implant prosthesis needs the marginal fit between the implant analog and the abutment to be precise. If the fit is correct, stress is more evenly handled and the holes in the bone are fully sealed [6]. Alternatively, an interface that is off by a little margin can result in mechanical faults, like some parts becoming loose, parts breaking, or the prosthetic not being stable enough [7]. After studying such disorders, it is known that they may bring about discomfort, uneven biting, and a higher chance of failing dental appliances [8]. Because of this, small gaps may become hotspots for infection, which can cause inflammation around the implant and slow down bone growth over time. Previous studies have examined the accuracy of impression materials, techniques for making molds and various ways to make casts, but we know less about the potential impact of repeated use of implant analogs compared to these other areas [9]. Existing research generally assumes that an implant would be used just once, but in many settings, reusing them is more practical [10]. This lack of information on the subject justifies further study, because very small changes in component sizes may influence how prosthesis performs and is adapted for use. Because of technological and patient changes in implant dentistry, knowing the tolerance levels of reusable parts becomes necessary. It is necessary to evaluate the accuracy of analog-abutment interfaces after using them several times to help guide decisions on when to change the components for safe clinical treatment. Therefore, it is of interest to check how many cycles of reuse change the dimensional accuracy and margin fit of implant analogs and abutments. It is hypothesized that using implant analogs over and over leads to gradual wear, making both the dimensions and margin adaptation vary greatly. The goal of the findings is to support setting limits for reusing implants, so reliable outcomes are possible with prosthetics.

## Materials and Methods:

This *in vitro* experimental study was conducted to assess the influence of multiple reuse cycles of implant analogs on their dimensional accuracy and the marginal gap with corresponding abutments.

## Sample selection and grouping:

A total of thirty titanium implant analogs (compatible with internal hex implant systems) were selected and randomly divided into three groups (n=10 per group) based on the number of reuse cycles:

[1] Group A: First-time use (control group)

[2] Group B: After 5 reuses

[3] Group C: After 10 reuses

Each reuse cycle consisted of embedding the analog into a type IV dental stone cast, attaching a prosthetic abutment and subsequently removing and cleaning the analog to simulate routine laboratory handling.

## Embedding procedure:

Analog specimens were positioned centrally in cylindrical silicone molds and secured using type IV dental stone under standardized conditions. After setting, abutments were connected to the analogs and tightened using a calibrated torque wrench at 30 Ncm, following manufacturer guidelines.

## Marginal gap measurement:

The assembled analog-abutment units were examined under a stereomicroscope (magnification 40x) to evaluate the marginal gap at four standardized reference points: mesial, distal, buccal and lingual. High-resolution digital images were captured and analyzed using Image J software to determine the vertical marginal discrepancies in micrometers (µm). The average of four readings per sample was calculated for statistical analysis.

## Dimensional analysis:

After each reuse cycle, the analogs were examined for dimensional accuracy using a digital caliper (±0.01 mm accuracy). Measurements were taken for analog diameter and thread depth. Surface wear or deformation was also visually inspected and recorded.

## Statistical analysis:

All recorded data were entered into SPSS software version 25.0. Descriptive statistics were computed for each group. One-way analysis of variance (ANOVA) was employed to detect significant differences among the groups, followed by Tukey,s post hoc test for pairwise comparison. A p-value less than 0.05 was considered statistically significant.

## Results:

The study evaluated the marginal gap and dimensional accuracy of implant analogs across three groups based on the number of reuse cycles. Results showed a progressive increase in marginal discrepancies and dimensional variations with repeated reuse. Group A (first use) exhibited the lowest mean marginal gap (23.4 ± 2.1 µm), followed by Group B (after 5 reuses) with 36.8 ± 2.7 µm and Group C (after 10 reuses) showing the highest gap of 52.1 ± 3.2 µm. One-way ANOVA indicated a statistically significant difference among the three groups (p < 0.001). Post hoc analysis revealed that differences between each pair of groups were statistically significant (p < 0.05) (Table 1 - see PDF). The diameter and thread depth of the implant analogs were measured after each reuse cycle. A gradual decrease in thread depth and analog diameter was noted with increased reuse. Group A had a mean analog diameter of 4.01 ± 0.02 mm and thread depth of 0.88 ± 0.01 mm, whereas Group C showed a reduced diameter of 3.95 ± 0.03 mm and thread depth of 0.81 ± 0.02 mm, indicating material wear and deformation (Table 2 - see PDF). These findings confirm that repeated reuse of implant analogs adversely affects both marginal fit and dimensional stability, which may compromise prosthetic accuracy (Tables 1 - see PDF and 2 - see PDF).

## Discussion:

Replicating the positions of the implants in the lab precisely is very important for long-term results with implant-supported prosthetics. In this process, implant analogs are important because they duplicate the design of the final implant fixture within the working model. Nevertheless, making many prosthetic sets from the same dental analogs might cause uncertainty about how soon the implant device will fail and how sharply the connections stay aligned. The study was designed to test whether repeated reusing played a role in changing the accuracy and fit of the margin area of analog-abutment assemblies. We determined that the marginal gap increases over each successive reuse cycle at a statistically significant level. The lowest marginal difference was in Group A, while repeating exposure led to the highest gap seen in Group C. The findings match what has been reported previously: that even slight problems in the fit of the bone can cause stress at the screws, leading these to become loose or the implant to be uncomfortable [1, 2]. In this study, the widespread presence of 50 µm or more gaps in the highly reused group is likely to cause bacterial microleakage and potentially result in peri-implantitis [4, 6]. Using dimensional analysis, we discovered a steady reduction in both the diameter and thread depth of the analog after every use, possibly due to wear and changes in the surface. Such alterations could reduce the correct fit of the prosthesis by lowering the strength and precision of how the implant anchors the crown [8].

It confirms other earlier studies' findings regarding the wearing of the components from frequent mechanical loading and tightening [9, 10]. The typical way to make implants durable is by using tough materials like titanium or stainless steel, but repeatedly applying force on the implant can still result in a distorted abutment-implant interface [11]. These tiny changes are usually undetectable without special tools, but they can still be noticed when studied at high resolution under the microscope [12]. In the present study, using a stereomicroscope and digital caliper, we observed slight but important differences in the dimensions as a garment was reused. It is common for laboratories to reuse implant analogs to save costs, but the literature is weak on finding out at what limit this might impact accuracy. Understanding the limit for safe reuse has been difficult; therefore, recommendations suggest reusing personal protective equipment at least 3 times, but at most 5, depending on how and with what the equipment is cleaned [13, 14]. This is also what our research shows, as mockups lose their tightness and shape accuracy as they are used more than five times. Paying attention to the torque applied when putting the abutments on the analog is also necessary. Uncontrolled torque may speed up wear on the analog and risk its precision [15]. We used a standard torque of 30 Ncm in our experiment to minimize this issue. Even so, using uncalibrated torque in real situations can lead to faster damage in the gear than predicted [16, 17]. An increase in the marginal gap has medical consequences as well. If passive fit is poor, the biomechanics of multi-unit restorations may become unbalanced and could fail [18]. Moreover, if analogs are the wrong size, this problem can be passed on to the prosthesis and influence occlusion, nearby tooth contacts and how satisfied the patient is. As a result, accurate analog components are necessary to maintain the success of dental restorations used in implants [19, 20]. This study does not directly consider factors such as saliva, the number of people who chew, and the microbial work found in the mouth. Performing *in vivo* or simulation studies with chew simulators may provide results more important to clinical care. Just one type of implant system was assessed, meaning performance results may not apply to every type of implant.

## Conclusion:

The use of implant analogs multiple times reduces their size accuracy and the quality of their connection to the abutment. Thus, analogs should be carefully watched and potentially changed after several reuses to ensure the curve of the prosthesis doesn't deviate.
